# Effects of Lower Limb Cycling Training on Different Components of Force and Fatigue in Individuals With Parkinson’s Disease

**DOI:** 10.3389/fbioe.2022.829772

**Published:** 2022-03-02

**Authors:** Yen-Po Lin, Rou-Shayn Chen, Vincent Chiun-Fan Chen, Chun-Hsien Liu, Hsiao-Lung Chan, Ya-Ju Chang

**Affiliations:** ^1^ School of Medicine, Chung-Shan Medical University, Taichung, Taiwan; ^2^ Department of Neurology, Chang Gung Memorial Hospital, Linkou, Taoyuan, Taiwan; ^3^ School of Medicine, College of Medicine, Chang Gung University, Taoyuan, Taiwan; ^4^ Neuroscience Research Center, Chang Gung Memorial Hospital, Linkou, Taoyuan, Taiwan; ^5^ Engineering Program, Loyola University Chicago, Chicago, IL, United States; ^6^ School of Physical Therapy and Graduate Institute of Rehabilitation Science, College of Medicine, Chang Gung University, Taoyuan, Taiwan; ^7^ Department of Electrical Engineering, College of Engineering, Chang Gung University, Taoyuan, Taiwan; ^8^ Healthy Aging Research Center, Chang Gung University, Taoyuan, Taiwan

**Keywords:** Parkinson’s disease, central fatigue, activation level, cycling exercise, low intensity exercise, fatigue, maximal voluntary contraction

## Abstract

The strength of lower extremity is important for individuals to maintain balance and ambulation functions. The previous studies showed that individuals with Parkinson’s disease suffered from fatigue and strength loss of central origin. The purpose of this study was to investigate the effect of lower extremities’ cycling training on different components of force and fatigue in individuals with Parkinson’s disease. Twenty-four individuals (13 males, 11 females, mean age: 60.58 ± 8.21 years) diagnosed with idiopathic Parkinson’s disease were randomized into training and control groups. The maximum voluntary contraction (MVC) force, voluntary activation level (VA), and twitch force of knee extensors were measured using a custom-made system with surface electrical stimulation. The general, central, and peripheral fatigue indexes (GFI, CFI, and PFI) were calculated after a fatiguing cycling protocol. Subjects received 8 weeks of low resistance cycling training (training group) or self-stretching (control group) programs. Results showed that MVC, VA, and twitch force improved (*p* < 0.05) only in the training group. Compared to the baseline, central fatigue significantly improved in the training group, whereas peripheral fatigue showed no significant difference in two groups. The cycling training was beneficial for individuals with Parkinson’s disease not only in muscle strengthening but also in central fatigue alleviation. Further in-depth investigation is required to confirm the effect of training and its mechanism on central fatigue.

## Introduction

Parkinson’s disease (PD) is a neurodegenerative disease and affects 1–2 per 1000 of the population at any time. PD prevalence increases with age, and PD affects 1% of the population above 60 years (Tysnes and Storstein, 2017). The typical features of individuals with Parkinson’s disease are bradykinesia, resting tremor, rigidity, and postural instability (Jankovic, 2008). In addition to the cardinal motor symptoms, there are also nonmotor symptoms, including fatigue, pain, sleep problems, autonomic nervous system problems, and cognitive problems. Among these nonmotor symptoms, fatigue is considered as an independent nonmotor symptom which appears early and persists throughout the disease course. A recent meta-analysis study showed that the prevalence of fatigue was up to 50% in PD (Siciliano et al., 2018). Fatigue was moderately associated with several negative health outcomes, such as apathy, anxiety, daytime somnolence, sleep disturbances, and poor quality of life (Siciliano et al., 2018).

The cause and the expression of fatigue are complicate. Studies showed that fatigue might be related to depression, but others showed fatigue also occurred in patients without depression (Finsterer and Mahjoub, 2014; Ferraz et al., 2018; Ghanean et al., 2018). According to the site of fatigue, fatigue can be divided into central and peripheral components. Central fatigue is attributed to the processes within the central nervous system (CNS) that reduce the neural drive to the exercising muscle and lead to a decrease in the voluntary activation (VA) level and subsequently its performance (Taylor et al., 2016; Sidhu et al., 2018). Peripheral fatigue, i.e., muscle fatigue, is attributed to neuromuscular transmission, excitation–contraction coupling, or muscle bioenergetics (Gandevia, 2001; Chang et al., 2008; Boyas and Guevel, 2011; Chen et al., 2014; Huang et al., 2017). In healthy aging subjects, the time to fatigue was longer; however, the percentage of central vs. peripheral fatigue was similar to young subjects (Mademli and Arampatzis, 2008). In the past, clinical measurements of fatigue in PD patients relied on subjective measurements and exercise-induced fatigue. However, our previous studies showed that exercise-induced fatigue had different impacts on central or peripheral origin fatigue and suggested that fatigue and muscle weakness in PD are pathologically central-originated which is different from fatigue in the normal aging process. PD patients suffered more central origin than peripheral origin muscle weakness and fatigue. This makes the alleviation of central fatigue in PD important (Huang et al., 2017). Since the mechanism of fatigue in PD is not similar to that of exercise-induced fatigue that occurs in non-PD population, strategies of alleviating fatigue, such as those suggested by the American College of Sports Medicine (ACSM), may not be suitable for PD-related fatigue. This would make a clinical prescription of the fatigue-alleviating program in PD patients distinctively different.

For fatigue in PD patients, especially central-originated fatigue, there is no clinical treatment with satisfactory results (Kluger et al., 2013). The effect of levodopa on fatigue is controversial (Schifitto et al., 2008). Exercise training is a common non-drug intervention for individuals with PD suggested in the study by Fisher et al. (2008), 8 weeks of treadmill training showed improvements in walking speed with a prolonged central silent period (CSP) observed. Alberts et al. (2011) showed that cycling training could produce a levodopa-like effect, which reduced tremors during off-stage. Cycling training also showed various beneficial effects on symptoms, such as tremor, cognitive function, and walking speed, in PD (Nadeau et al., 2016) even with low intensity (Chang et al., 2018). These studies showed that cycling exercise is beneficial to PD patients. However, whether cycling training altered the fatigue status, especially the central origin fatigue, is not clear.

Cycling training is a common endurance training for healthy populations, which could increase joint mobility, muscle strength and endurance, prevent muscle atrophy, and improve cardiorespiratory fitness (Bourne et al., 2018; Ferraz et al., 2018; Silveira et al., 2018; Chavarrias et al., 2019; Ahmed and Babakir-Mina, 2021). Cycling training also has an improved effect on patients with central fatigue. For patients with multiple sclerosis, 8 weeks of cycling training improved not only the walking speed but also the maximum exercise times (Cakt et al., 2010). Cycling training is a potential strategy. However, none of the previous studies has reported success in alleviating Parkinson-related central fatigue. This was probably due to the cycling protocol, in terms of the resistance level and dosage, which was not designed specifically for central fatigue.

In exercise training, the training program has to be specific and relevant to the targeted activities or sports in order to produce the desired effect. Our recent study showed that the level of resistance of cycling exercise is critical in the mechanisms of fatigue. With an equivalent dosage, cycling at a lower resistance level could challenge the center fatigue-related mechanism more (Hsu et al., 2020). It is plausible to hypothesize that cycling training in this central fatigue challenging resistance could alleviate Parkinson-related fatigue, but its evidence has never been obtained. In addition, whether the alleviation of central fatigue was related to the severity of subjective fatigue of PD patients was not clear. Therefore, the purpose of our study employed a randomized control design to evaluate the effect of cycling training with a central fatigue challenge resistance on individuals with PD. In order to identify the candidate most likely to benefit from cycling training, our secondary purpose is to evaluate the correlation between the training gain, if there is any, and the baseline feature of patients.

## Materials and Methods

### Participants

Twenty-four individuals (13 males, 11 females, mean age: 60.58 ± 8.21 years) diagnosed with idiopathic PD, according to the United Kingdom Brain Criteria, were recruited from the outpatient clinics. The sample size was estimated by G*Power software according to the pilot study with the effect size f = 0.5, *α* = 0.05, and power = 80%. Considering the potential dropout rate, twenty-four subjects were recruited. The inclusion criteria include 1) Hoehn and Yahr stages II-III, 3) stable medication usage, and 4) mini-mental state examination score ≥24. Patients who had tremors when on medication or during recording and those with other central or peripheral neurological diseases or musculoskeletal injuries of the lower limbs were excluded from the study. Included individuals were further randomized into training and control groups (Table 1). All tests and training were performed during the clinical “ON” status. Written informed consent was obtained before participation. This study was approved by the Institutional Review Board.

**TABLE 1:** The characteristics of subjects.

	Training	Control	*p* value
Gender (M:F)	8:4	5:7	—
Age (yr)	61.5 ± 5.65	60.58 ± 8.21	0.05
H&Y (score)	1.625 ± 0.33	1.42 ± 0.36	0.71
Duration (yr)	5.5 ± 1.73	5.50 ± 2.28	0.29
MoCA (score)	27.17 ± 0.94	27.75 ± 0.62	0.25
FSS (score)	5.18 ± 0.68	4.70 ± 0.55	0.28

### Baseline Evaluation

After inclusion, the basic data including demographic data (age and gender), Hoehn and Yahr stages, MoCa, fatigue severity scale (FSS), and walking speed were obtained for post-training comparison. FSS (Herlofson and Larsen, 2002) is a questionnaire commonly used to assess patients’ subjective grading of fatigue on their daily living.

Subjects then moved to a stationary bike with a custom-made knee extension force measurement system with a force transducer (AWU, Genisco Technology, CA, United States) coupled to a transducer amplifier (Gould Inc., Valley View, OH, United States). Data were sampled at 1000 Hz *via* (InstruNet Model 200 PCI controller, United States) and recorded on a computer for offline analysis. This system can measure the knee isometric extension force at 90 degrees of flexion after biking without changing the position with a good reliability. For further details, please refer to the previous study (Hsu et al., 2020).

Electrical stimulation (stimulator model DS7A, Digitimer Ltd.; Hertfordshire, United Kingdom) with surface electrodes (9 × 12 cm) was used for measuring the VA and twitch force of quadriceps. The electrodes were placed on the muscle belly of the quadriceps. The induced force was monitored simultaneously with an oscilloscope (TDS220, Tektronix Inc., Beaverton, OR, United States). The force signal was digitized using an analog-to-digital converter with 16-bit resolution (InstruNet Model 200 PCI controller, United States) at 1000 Hz.

**FIGURE 1:**
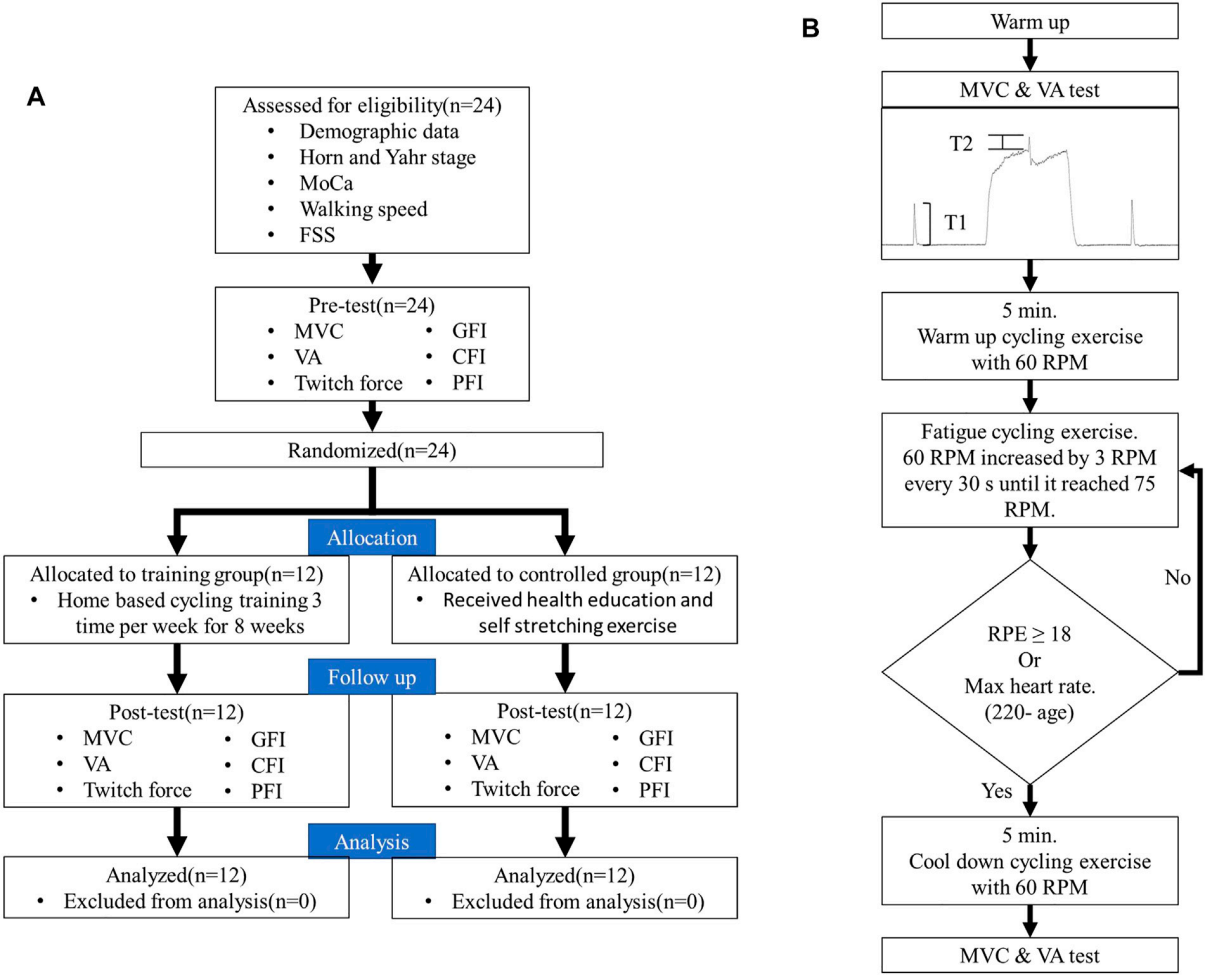
Flowchart of the study **(A)** and the protocol **(B)**.

After the warm-up contractions, participants performed three maximal voluntary contractions (MVCs), each sustained for 5 s. After the MVC test, the VA was evaluated by the interpolated twitch technique (ITT) (Figure 1A). During this test, the supramaximal electrical stimulation (200 μs duration with 120% of the maximum intensity) was applied under relax and during knee extensor MVCs to obtain the resting twitch (*T*_*1*_) and interpolated twitches (*T*_*2*_). The resting twitch represents the peripheral component of force whereas the interpolated twitch represents the force generated from spared motor units that failed to be activated by the CNS (Huang et al., 2010; Chang et al., 2011; Huang et al., 2017; Chuang et al., 2019; Tang et al., 2020). The resting twitch was averaged from the twitch elicited before and after the MVC to avoid the variation caused by force potentiation. The VA was calculated by the following formula.

$$VA=\left(1-T_{2}/T_{1}\right)\times 100\%.$$

### Cycling Protocol for Inducing Fatigue

**FIGURE 2:**
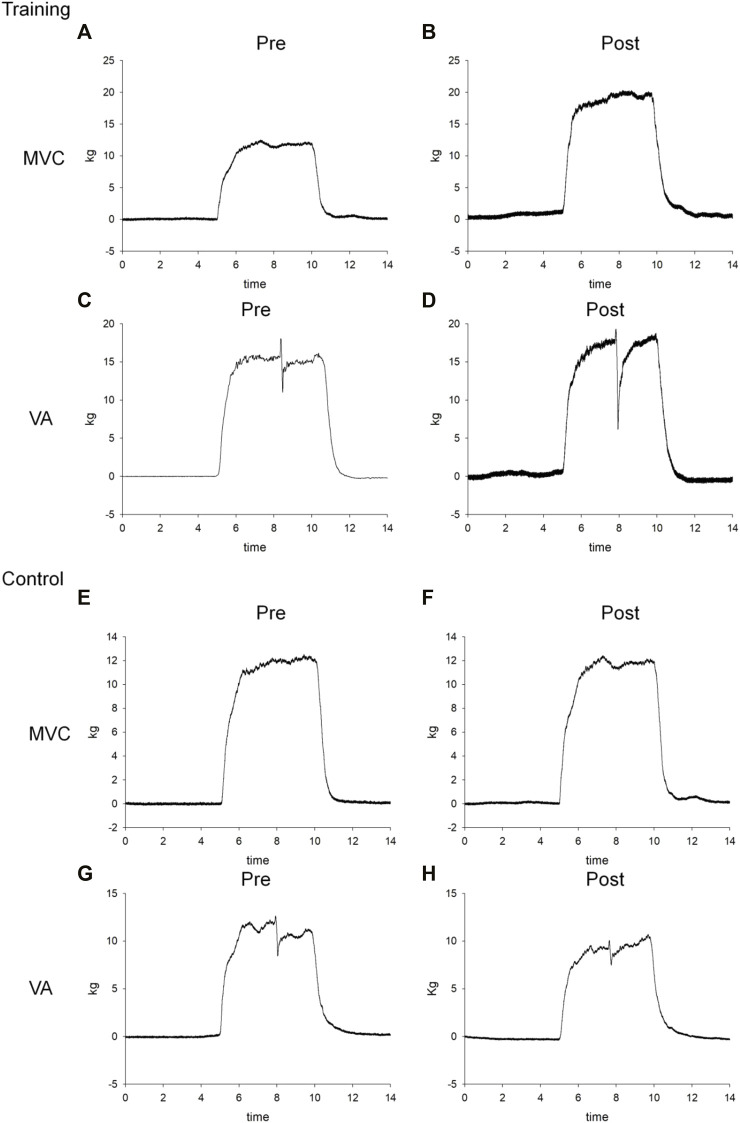
Representative force–time curves of the MVC and VA for one training patient **(A–D)** and one control subject **(E–H)** before (pre) and after (post) 8-week cycling training.

After the measurements of MVC, VA, and twitches, subjects received a cycling protocol to induce fatigue. The cycling protocol was adapted from the previous study with an intensity found to predominantly induce central fatigue (Hsu et al., 2020). This protocol included a 5-min warm-up by cycling at 60 revolutions per minute (RPM) with no resistance. After warming up, the cycling speed increased by 3 RPM every 30 s until it reached 75 RPM. The target resistance was 70 W, which corresponded to 25% MVC. During the testing session, the rate of perceived exertion (RPE) was reported by the participants every 1 min. Once the participants’ RPE reached 18 or the participants’ maximum heart rate (maximum = 220 minus the age), the cycling test was terminated, and 5 min of cool-down was provided (Figure 1B). The parameters of the cycling protocol were recorded for each subject for the ensuing training protocol and for post-training testing. This protocol complied with the exercise guideline for safety suggested by the ACSM to prevent blood pooling in lower extremities and facilitate venous return (American College of Sports Medicine, 2016). After the cycling protocol, MVCs, VA, and resting twitches were measured again to evaluate general fatigue, central fatigue, and peripheral fatigue (Figure 1). Representative data for the MVC and interpolated twitch force are shown in Figure 2.

The ratio of post-fatigue MVC to pre-fatigue MVC was calculated as the general fatigue index (GFI). The ratio of the post-fatigue VA to pre-fatigue VA was calculated as the central fatigue index (CFI). The ratio of the post-fatigue twitch force to the pre-fatigue twitch force was calculated as the peripheral fatigue index (PFI) (Chien et al., 2008; Ju et al., 2011). A higher fatigue index indicates less fatigue.

### Training Protocol

Subjects in the training group then received home-based cycling training three times per week for 8 weeks. The intensity and speed were determined individually based on the baseline cycling protocol described previously. Subjects in the control group received health education and self stretching exercise.

One week after the last training, the subjects returned to the laboratory for post-training evaluation of the MVC, VA, twitch, GFI, CFI, and PFI. The evaluation procedures were the same as in baseline evaluation.

### Data Reduction and Statistical Analysis

Two-way (group by time) repeated measures of ANOVA with the *post hoc* Tukey test were used to analyze the MVC, VA, twitch force, GFI, CFI, and PFI. Once a significant interaction was detected, one-way ANOVA was applied for individual groups. The Spearman correlation coefficient was used to estimate the correlation between the training-induced gain and the baseline condition of patients. The significance level was set at *p* < 0.05. Statistical analyses were performed using SAS (version 9.4; SAS Institute, Cary, NC, United States).

## Results

### Strength

**FIGURE 3:**
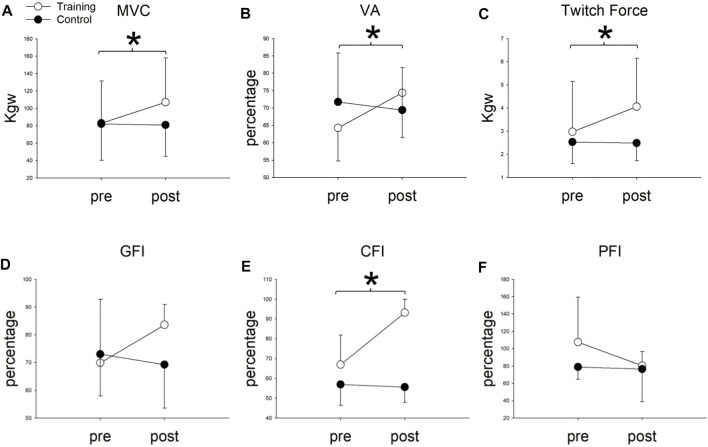
**(A)** Maximal voluntary contraction (MVC), **(B)** voluntary activation (VA), **(C)** twitch Force, **(D)** general fatigue index (GFI), **(E)** central fatigue index (CFI), and **(F)** peripheral fatigue index (PFI) before (pre) and after (post) 8-weeks cycling training between training and control groups. *Significant difference between pre- and post-8-week cycling training in the training group (*p* < 0.05).

Two-way repeated measures of ANOVA showed significant group x time interaction in the MVC, VA, and twitch forces (Table 2). In the training group, the MVC, VA, and twitch force significantly increased from 82.96 ± 48.69 to 107.17 ± 50.75 kg (*p* = 0.002), 64.32 ± 9.60 to 74.36 ± 12.85% (*p* = 0.046), and 2.97 ± 2.17 to 4.06 ± 2.09 kg (*p* = 0.001) after 8 weeks of cycling training, respectively (Figure 3). In the control group, none of the MVC, VA, or twitch changed after 8 weeks (*p* > 0.05).

**TABLE 2:** The mean, standard deviation, and results of ANOVA of the maximal voluntary contraction (MVC), voluntary activation level (VA), twitch force, central fatigue index (CFI), peripheral fatigue index (PFI), and general fatigue index (GFI) before (pre) and after (post) 8-week cycling training in two groups. The main effect is not shown if the interaction is significant (*p* <0 .05).

	Pre (mean ± standard deviation)		Post (mean ± standard deviation)		2 way ANOVA (*p*-value)		
	Training	Control	Training	Control	Interaction	Main effect time	Main effect group
MVC	82.96 ± 48.69	82.32 ± 41.86	107.17 ± 50.75^a^	80.99 ± 36.09	0.0006^b^	—	—
VA	64.32 ± 9.60	71.75 ± 14.11	74.36 ± 12.85^a^	69.40 ± 12.19	0.0194^b^	—	—
Twitch force	2.97 ± 2.17	2.53 ± 0.93	4.06 ± 2.09^a^	2.49 ± 0.77	0.0027^b^	—	—
CFI	66.96 ± 14.88	57.02 ± 10.63	93.21 ± 6.70^a^	55.68 ± 7.81	0.0001^b^	—	—
PFI	107.69 ± 51.74	78.88 ± 14.12	80.46 ± 16.23	76.57 ± 37.56	0.2162	0.1454	0.1057
GFI	69.96 ± 22.88	73.08 ± 15.07	83.63 ± 7.31	69.31 ± 15.78	0.08	0.3089	0.2376

a Significant (*p* < 0.05) difference in comparison to pre-training conditions.

b Significant interaction (*p* < 0.05) between time and group.

This suggested that the muscle force increased in both central and peripheral origins after training. In contrast, the muscle forces did not change in the control group (*p* > 0.05) after 8 weeks.

### Fatigue

For fatigue indexes, two-way repeated ANOVA showed significant group x time interaction only in the CFI (*p* < 0.001) but not in the GFI (*p* = 0.08) or PFI (*p* = 0.216). Compared to the baseline, the CFI significantly increased from 66.96 ± 14.88 to 93.21 ± 6.70 (*p* = 0.0001) in the training group, whereas the CFI did not change in the control group. (CFI = 57.02 ± 10.63 and 55.68 ± 7.81 before and after 8 weeks, *p* = 0.72). For the GFI and PFI, no group x time interaction (*p* > 0.05), or main effect in time (*p* > 0.05) or group (*p* > 0.05), was found. These results indicated that cycling training minimized the degree of central fatigue (Table 2) (Figure 3).

### Correlation With the Baseline Status

**FIGURE 4:**
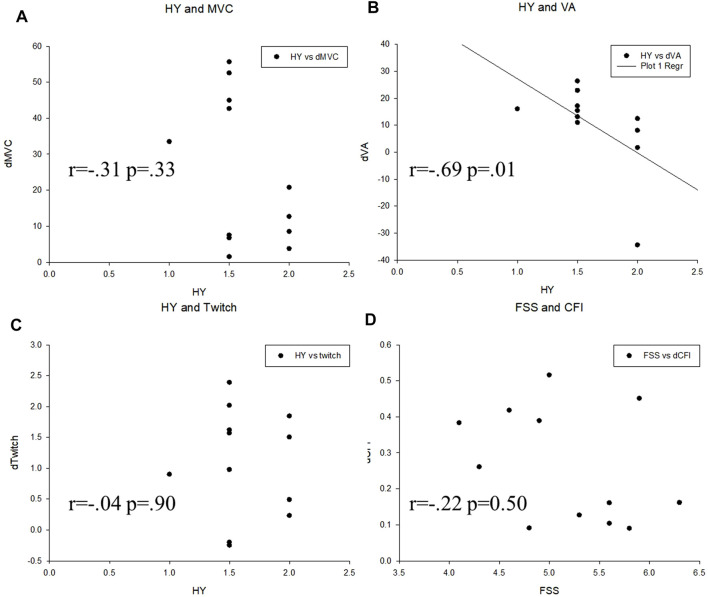
Correlation analysis between variables in the two groups. **(A)** Hoehn and Yahr scale (HY) and maximal voluntary contraction (MVC), **(B)** Hoehn and Yahr scale (HY) and voluntary activation (VA), **(C)** Hoehn and Yahr scale (HY) and twitch force (Twitch), **(D)** Fatigue severity scale (FSS) and central fatigue index (CFI).

The Spearman correlation coefficient showed that the Hoehn and Yahr stage has moderate negative correlation with training-induced improvement in the VA (r = −0.69 *p* = 0.01) but not the MVC (r = −0.31 *p* = 0.33) or twitch force (r = −0.04 *p* = 0.9). The CFI improvement was not correlated with the baseline status of FSS. (r = −0.22 *p* = 0.50) (Figure 4).

## Discussion

We conducted a randomized control trial investigating the effects of cycling training on 24 PD patients who were classified into the training and control groups. The main finding was that using a central fatigue-challenging intensity, the cycling training group improved in the MVC, VA, and twitch force more than the control group in patients with PD. Furthermore, a significant difference in the CFI index was found between the two groups after intervention. To our knowledge, this is the first study to prove that a cycling training at a central fatigue-challenging resistance could improve strength and alleviate central fatigue in individuals with PD. In addition, the training effect is better for earlier stage PD patients, but not influenced by the severity of subjective fatigue in the baseline.

Bicycle endurance training is a common treatment in clinical practice. Its main functions include maintaining or increasing joint mobility, muscle strength and endurance, preventing muscle atrophy, and improving walking function (Kubukeli et al., 2002; Katz-Leurer et al., 2006). Although it is already known that bicycle training improves the motor function of patients with PD, its neural mechanism and how training affects the fatigue of patients with PD are not clear. Fatigue management of PD is always a challenge for clinicians partially due to the complicated etiology per se (Kostic et al., 2016). The reason why previous studies failed to show the effect of training intervention on fatigue in PD may be due to its complicated etiology and the clinically subjective measurements. The subjective fatigue measurement required individuals to sensibly respond to the feeling of fatigue which would be influenced by the subjects’ other daily activity levels. It only reflects subjective aspects of the effects of fatigue on subjects’ daily activities. Therefore, the aim of our study is to offer more objective and quantitative results. Since PD is a long-term degenerative disease, early intervention and patients’ adherence to continuing exercise regimen are essential (Salgado et al., 2013). Therefore, different from previous studies, milder PD patients with stable medication were included in our study (Ridgel et al., 2011).

The major finding in our study, as predicted, is that the MVC, VA, and twitch force improved in the training group. These findings agree with previous studies indicating that PD patients whose proprioceptors are continuously activated by riding a bicycle may be vital for the recovery of motor functions (Ridgel et al., 2015). There were no previous studies that reported improved central fatigue by cycling exercise in PD patients. Although the etiology of central fatigue in patients with multiple sclerosis is not identical to that in PD, Cakt et al. (2010) showed that after 8 weeks of cycling training for patients with multiple sclerosis, the exercise tolerance increased, and the subjective fatigue feelings decreased. Both are regarded as indicators of fatigue alleviation. Our study not only provided evidence of cycling training in PD patients but also specified the training effects on central and peripheral components of lower extremity strength and central origin fatigue. Alberts et al. (2011) offered more information regarding the effects of 8-week bicycle training intervention on slowing down the progress of PD. In our study, the subjects were under medication control while showing improvements through cycling training. This indicates that cycling training improves motor function in patients with PD concomitantly under medication.

We further use the CFI and PFI to explore and compare the changes in central fatigue and peripheral fatigue before and after cycling training intervention in patients with PD. Notably, the intervention group had significantly better improvement than the control group (Figure 3E) in the CFI. It is known that peripheral fatigue is produced by changes at nerve branches, neuromuscular junction, or distal to these structures. Central fatigue originates from the central nervous system, which decreases the neural drive to the muscle (Davis, 1995). Currently, it is recognized that central fatigue is more pronounced in PD patients, which means that the alleviation of central fatigue can possibly enhance the functional activities of daily living in PD patients (Huang et al., 2017). Ferraz et al. (Juvet et al., 2017; Ferraz et al., 2018) have also outlined the high prevalence of subjective fatigue in PD patients. Our study is novel in using an RCT design to show the effect of cycling training, especially on central fatigue in PD patients. The result may apply to other diseases, such as joint hypermobility syndrome and multiple sclerosis that share similar central fatigue symptoms (Chang et al., 2011; To et al., 2019).

The fatigue of PD patients is puzzling and poorly understood. There are several mechanisms which have been proposed to explain improvements related to physical training. Bicycle training on PD patients with mild-to-moderate severity is reported to have better activation in the putamen of the basal nucleus and the globus pallidus (Alberts et al., 2011). Similar studies also report coordination training or motor fitness training to be promising means to increase the basal ganglia volume which is related to coordinative aspects of fitness and partially explained the cognitive performance (Niemann et al., 2014). Other proposed mechanisms, for example, the brain-derived neurotrophic factor (BDNF), which is induced by aerobic exercise, may possibly explain the relationship between central fatigue and cycling training. The BDNF not only enhances synaptic GABA clearance (Boyne et al., 2019) but also potentiates normal central nervous system myelination in development and enhances recovery after myelin injury (Fletcher et al., 2018). Another possible and direct reason may be that central fatigue has been suggested to be related to neurotransmitters such as serotonin and dopamine (Dobryakova et al., 2015). In previous studies, dopamine has been shown to increase during exhausting exercise (Fisher et al., 2004; Petzinger et al., 2007), and a reduced level of dopamine was reported in fatigued rats (Meeusen et al., 2010). In addition, Petzinger et al. have shown that exercise may influence activity-dependent processes in the basal ganglia through alterations in dopaminergic neurotransmission and have demonstrated that exercise-induced behavioral benefits may be due to changes in cortical hyper-excitability normally observed in the dopamine-depleted state (Petzinger et al., 2010). Although the afore mentioned studies provide basic evidence, the unclear mechanisms of PD-related central fatigue phenomena still require further studies to investigate the potential interaction between exercise, neurotransmitter, and neuronal activation.

Our study showed that the training-induced gain were not correlated with the baseline FSS, suggesting that patients in different subjective statuses would be benefitted from this training. However, the negative correlation between the improvement of the VA to Hoehn and Yahr stages suggests that the training effect would be better in earlier stages. Patients are suggested to receive training in earlier stages to receive better effects.

There are some limitations while applying the results of this study in clinical settings. First, only patients with Hoehn and Yahr stages II-III with stable medication were enrolled in this study. The optimal training setting for more advanced PD patients remains to be investigated. Second, to avoid interference of tremors on the resting twitch force measurement, subjects with obvious tremors and subjects who had tremors during recording were excluded. Enhanced twitch activation during muscle training was merely recorded for analysis. The training effects on tremor-dominant patients require future in-depth investigation. Finally, we did not use gender stratification when recruiting. Based on previous studies that have compared fatigue between elderly women and men, there is no significant difference in gender (Hicks and McCartney, 1996; Ekman and Ehrenberg, 2002). Thus, we consider that gender differences are not a significant interfering factor in our study. Future studies with gender stratification design are suggested to further clarify the potential gender issue.

## Conclusion

The present study confirmed that cycling training is efficient, beneficial, and feasible for patients with early-stage PD in strengthening both central and peripheral components of knee extensor forces. Moreover, this is also the first study to show that cycling training at a low resistance alleviates central fatigue, especially for patients with pronounced central fatigue. The training effects could be shown on individuals with PD who have different baseline levels of subjective fatigue. These findings provide important insights which will be useful for the development of rehabilitation interventions for PD and suggest that progressive cycling training could serve as a new treatment for early-stage PD.

## Data Availability

The raw data supporting the conclusion of this article will be made available by the authors, without undue reservation.
